# The Safe Pregnancy study - promoting safety behaviours in antenatal care among Norwegian, Pakistani and Somali pregnant women: a study protocol for a randomized controlled trial

**DOI:** 10.1186/s12889-019-6922-y

**Published:** 2019-06-10

**Authors:** Lena Henriksen, Eva Marie Flaathen, Jeanette Angelshaug, Lisa Garnweidner-Holme, Milada Cvancarova Småstuen, Josef Noll, Angela Taft, Berit Schei, Mirjam Lukasse

**Affiliations:** 1 Department of Nursing and Health Promotion, Oslo Metropolitan University, St. Olavs plass, P.O. Box 4, 0130 Oslo, Norway; 2 Department of Technology Systems, University of Oslo, P.O box 20, 2007 Kjeller, Norway; 3 Judith Lumley Centre, La Trobe University, Bundoora, Melbourne, VIC 3086 Australia; 4 Department of Public Health and General Practice at the Faculty of Medicine, The Norwegian University of Science and Technology (NTNU), Håkon Jarls gate 11, N-7489 Trondheim, Norway; 5 Department of Gynaecology at the Women’s Clinic, St. Olavs Hospital, Trondheim University Hospital, Sluppen, Postbox 3250, N-7006 Trondheim, Norway

**Keywords:** Intimate partner violence, Antenatal care, Tablet technology, Video intervention; culture sensitivity

## Abstract

**Background:**

Intimate partner violence (IPV) around the time of pregnancy is a recognized global health problem with damaging consequences. However, little is known about the effect of violence assessment and intervention during pregnancy. We hypothesise that routine enquiry about IPV during pregnancy, in combination with information about IPV and safety behaviours, has the potential to increase the use of these behaviours and prevent and reduce IPV.

**Methods:**

The Safe Pregnancy study is a randomised controlled trial (RCT) to test the effectiveness of a tablet-based intervention to promote safety behaviours among pregnant women. Midwives include women who attend routine antenatal care. The intervention consists of a screening questionnaire for violence and information about violence and safety behaviours through a short video shown on a tablet. The materials are available in different languages to ensure participation of Norwegian, Urdu, Somali and English-speaking women. Eligible women answer baseline questions on the tablet including the Abuse Assessment Scale (AAS). Women who screen positive on the AAS will be randomized to an intervention video that contains information about violence and safety behaviours and women in the control group to a video with general information about a healthy and a safe pregnancy. All women receive information about referral resources. Follow up will be at three months post-partum, when the woman attends the maternal and child health centre (MCHC) for the baby’s check-up. Outcome measures are: Use of safety behaviours and quality of life (primary outcomes), prevalence of violence, mental health measures and birth outcomes (secondary outcomes). Intention to treat analysis will be performed.

**Discussion:**

The project will provide evidence on whether enquiry about violence and a short video intervention on a tablet is effective and feasible to prevent or reduce harm from IPV among women who attend antenatal care.

**Trial registration:**

This study is registered in ClinicalTrials.gov. Identifier: NCT03397277 (Registered 11th January 2018).

## Background

The World Health Organization (WHO) states that 30% of women worldwide have been exposed to physical and/ or sexual violence by intimate partner sometimes during life [1]. Pregnancy does not protect women from violence and the prevalence indicates that health professionals will meet women that are or have been exposed to intimate partner violence (IPV) when caring for pregnant women. A meta-analysis of IPV during pregnancy consisting of 92 studies from 23 countries, reported an average prevalence of emotional abuse of 28.4%. The prevalence of physical abuse and sexual abuse was 13.8 and 8.0% respectively [2]. In Norway, the prevalence varies from one to 5 % in different studies [3–5]. These numbers are comparable with a longitudinal cohort study from Sweden [6]. Among 1573 women in this study, 2.5% reported violence during pregnancy [6]. The prevalence increased in the early postnatal period to 3.3% [6]. The Norwegian studies were not conducted among minority populations and a knowledge gap exists regarding IPV in different immigrant groups. Although IPV occurs in all social strata, women with low education and women with limited economic resources are at higher risk [7]. Immigrant women are likely to be overrepresented in these groups; hence they are more prone to be exposed to IPV [7]. In Norwegian crisis shelters, immigrant women are overrepresented [8]. Therefore, an increase in our awareness of cultural sensitivity, also when delivering health care services and developing interventions, is required [9].

IPV prior to pregnancy, during pregnancy or in the new-born period is associated with adverse health outcomes like depression, miscarriage, stillbirth, preterm birth and low birth weight [1, 3, 4, 10–15]. It may also affect motherhood and the way women interact and connect with their babies [16]. In Norway, more women die at the hand of their partner/former partner, than from pregnancy and birth related complications [17, 18].

Antenatal care is recognised as an ideal ‘window of opportunity’ to address IPV because this is a time when women are in regular contact with heath care providers [19]. Pregnancy is an important context for safety planning as child well-being and safety is a priority for many abused women [20]. Almost all pregnant women in Norway attend antenatal care that is free of cost [21]. Women can choose to go to a midwife or a family doctor or alternate between both for antenatal check-ups, thus midwives have a central role in antenatal care [21] and an opportunity to identify women at risk and provide them with the help they need. Studies suggest that screening for IPV in antenatal care is likely to increase identification of violence [22]. Guidelines in Norway instruct health professionals to routinely ask all pregnant women about their experience of violence [23]. Recommended interventions for IPV in primary care settings involve questions about violence, information of safety-promoting behaviours, and referral to community resources [24, 25]. However, the evidence regarding how to assess and intervene against violence during pregnancy and the new-born period is inconclusive [10, 26–28] and there is a lack of evidence of effective interventions [29].

The aim of the Safe Pregnancy study is assisting midwives in antenatal care to enquire about violence by providing them with additional education. In addition, we will develop a culturally sensitive intervention [9] in collaboration with women who have been pregnant and have suffered violence, as well as consulting health care professionals. The tablet-based intervention will include a screening tool for violence and a video that promotes culturally adapted safety behaviours for women who read and speak Norwegian, English, Urdu or Somali. Our intervention of staff training, a screening tool, and a safety-promoting video aims to reduce harm from violence.

## Methods

### Study design

The Safe Pregnancy study is a randomized controlled trial (RCT) to test the effectiveness of a tablet-based intervention that promotes safety behaviours. It includes four stages (described in more detail below): 1) Development of the questionnaire including a screening tool for violence and safety-promoting video (intervention) 2) Professional development for midwives regarding IPV 3) Conduct of the RCT within antenatal care 4) Process evaluation.

### Objectives

The objectives of this study are to:

1. Select a screening tool for violence and develop an intervention to increase safety behaviours, culturally and linguistically adapted to Norwegian, Somali and Pakistani women.
2. Increase the knowledge among midwives regarding IPV and their skills in enquiring and caring for women who are exposed to violence.
3. Assess the level of IPV during pregnancy before and after the intervention among Norwegian, Pakistani and Somali women.
4. Assess the effect of the intervention through a RCT.
5. Explore pregnant women’s experience of the intervention.
6. Evaluate midwives experience of the study with the tablet technology.

**Fig. 1:**
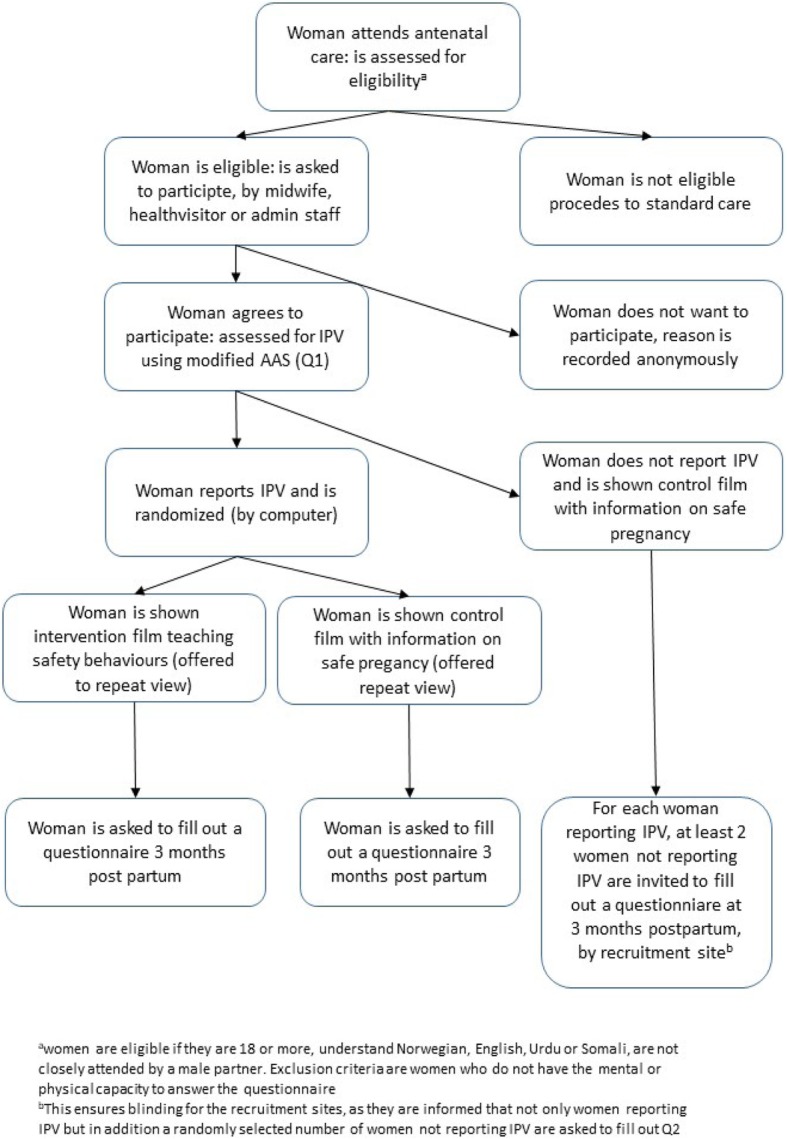
The study flow chart

The Safe Pregnancy RCT will be carried out in a routine antenatal care setting at 19 maternal and child health centres (MCHC) in South-Eastern Norway. The study will be introduced as one that examines different aspects of having a safe pregnancy, including stress, quality of life, and IPV. This is to emphasise the focus on staying healthy and safe during the pregnancy and be able to mask the intervention to the women. Following informed consent, individual women will be randomized to the intervention or control group. The women in the intervention group will see a seven-minute video containing information about violence and safety behaviours, women in the control group will see a seven-minute video with information about a healthy and safe pregnancy in general (e.g food, smoking, alcohol, physical and mental health), including information about referrals for violence. All participating women will get an appointment card with a list of phone numbers and websites to governmental and local resources that promote a safe pregnancy as well as the number for police and ambulance services at the back. Follow up will be at 3 months post-partum, when the woman attends the maternal and child health centre (MCHC) for the baby’s check-up. The data collection will be in compliance with Consolidated Standards of Reporting Trials (CONSORT) guidelines [30]. Figure 1 shows the study flow chart.

### Participant inclusion criteria

Pregnant women ≥18 year, at any gestational age who understand Norwegian, Urdu, Somali or English at the participating MCHC, will be screened for eligibility. Women who cannot be screened without their partners or other family members and women who do not have the mental or physical capacity to answer the questions will be excluded. The rationale for choosing the specific immigrant groups is that Pakistani and Somali communities are among the largest immigrant groups in Norway with high fertility rates [31]. Both countries of origin have patriarchal norms that may permit IPV [32]. In addition, Somali immigrants are regarded as a group with potential previous exposure to interpersonal violence due to the prolonged conflict situation in Somalia [33].

### Sample size

Little is known about our study population regarding the use of safety behaviours and there is a lack of information in the literature about anticipated mean values and standard deviation (SD) for the measurements before and after the intervention. Moreover, little is known about what would be considered a clinically meaningful change. Therefore, the power calculation is based on a ‘worst case scenario’ with low number of safety behaviours at baseline and a small change after the intervention. We do not anticipate any change before and after for the control group. Thus, using McNemar’s test for correlated proportions as the same women are interviewed before and after the intervention, we anticipate that the proportion before would be 20% (3 behaviours out of 15) and this proportion would increase to 30% (5 safety behaviours) for the intervention group. Further assuming the responses to be moderately correlated (0.6) we would need 75 women in both groups to keep power of 87% and alpha of 5%. If the correlation is weaker (0.5) we would have power of 80% given the same sample size. So with 75 women in both groups we consider our study to be sufficiently powered.

### Recruitment and randomization

All eligible women will be recruited to the trial by their midwife during routine antenatal check-ups at the MCHC. Women will be asked to answer baseline questions on a tablet. A modified version of The Abuse Assessment Screen (AAS) is part of the baseline questionnaire. AAS is a five-item screening tool that has been tested in obstetrics–gynaecology outpatient practices and among different ethnicities [34, 35]. Exposure to violence will be determined by a positive response to at least one of the five questions in AAS. Specifically, they will be asked: Have you ever been afraid of your partner or someone else? Have you ever experienced that a partner or ex-partner has: Done things to make you feel afraid of them? Done things to try to intimidate you or to control your thoughts, feelings or actions? Hit, kicked, pulled you by your hair or otherwise physically hurt you? Forced you to have sexual activities against your will? The answer options are: Never, Yes previously, Yes during the past 12 months before the pregnancy, Yes, since the start of the pregnancy.

After completion of the baseline questionnaire, women who screen positive will be randomized by block randomization at the tablet level to either the intervention or the control video. All women who screen negative on the AAS will see the control video. Women will not be informed if the video is the intervention or control video. The midwives will be blinded to which video intervention the women receive. Unless midwives ask women about the content of the video or the woman discloses this, they will not know which video participants have viewed. Investigators who perform the outcome analysis in Safe Pregnancy will be blinded to the allocation of participants until after the analysis.

### The intervention

Using tablet technology is a new and original approach in antenatal care. The screening tool for IPV and other questions for the baseline data collection will be developed for use on the tablet. The tablets allow uniformity of the data-collection and less opportunity for research bias. Women will be able to select among Norwegian, Urdu, Somali, English and can move from one to another language if needed while filling out the questionnaire.

The safety-promoting video, which women randomized to this option will view right after the questionnaire, will consist of digital storytelling: combining narrative with digital content, including images, sound, and video that focus on IPV and safety behaviours. Digital storytelling has been advocated as a strategy to empower people and facilitate learning [36]. With the help of initial qualitative user-involvement studies, the video will be culturally and linguistically adapted to Pakistani, Somalian and Norwegian women. The safety behaviours from McFarlane will be adopted to a Norwegian setting and women will be encouraged to talk to their midwife if they do not feel safe. All women will be informed that they can view their video as many times as they want during pregnancy while visiting the MCHC. They can access the video by using their study number.

In the adaptation of the screening tool and development of the safety-promoting video will make use of several resources: User participation, IPV in primary care guidelines [23, 26], systematic reviews of health-care based interventions [29] and studies documenting inter-cultural communication [9, 37].

We will undertake a User involvement study with a qualitative exploratory design as part of stage one. The data will be collected through individual in-depth interviews with Norwegian women and women with Pakistani and Somali ethnic background, both previously exposed and not exposed to IPV. In addition, focus group interviews will be conducted with skilled professionals at Norwegian crisis shelters.

### Professional development of the midwives

All participating midwives were invited to an international conference about violence during the time of pregnancy before the project started. Shortly after we had an 8-h day only for the participating midwives that included presentations, participatory activities and reflections regarding violence against pregnant women and the project. All MCHCs will get individual teaching sessions in the use of the tablet, how to assess eligibility and recruit women. Project meetings with a mix of presentations from different resources within the field and reflections will be held throughout the recruitment period.

### Outcomes

The primary outcomes are:

- Use of safety behaviours: The list of 15 safety behaviours was developed by Mc Farlane et al. [38–40]. The list is adapted to a Norwegian setting (Table 1). It still consists of 15 safety behaviours that women will be asked to consider. The answering options are yes, no, not applicable. The sum score is computed and adjusted for number of not applicable answers as follows:
  x = 15 * (a/b) where a/b is the proportion of recognized safety behaviours out of the number of applicable behaviours. Thus, the adjusted total falls between 0 and 15. The equation used to calculate the adjusted total is: *a*/*b* = *x*/15, where a is the number of behaviours performed, *b* is the number of behaviours applicable, and x is the adjusted total. When *a* and *b* are known the adjusted total number can be calculated by cross-multiplying the two fractions. Our hypothesis is that an increase in the numbers of safety promoting behaviours is positive.
- World Health Organization Quality of Life – Bref (WHOQOL-BREF): Quality of life will be measured with the WHOQOL-BREF [41]. The WHOQOL-BREF is an abbreviated 26-item version of the WHOQOL-100. The WHOQOL-BREF is a shorter version of the original instrument and more convenient to use in large research studies or clinical trials. It consists of two global items on overall quality of life and general health, and four domains: Physical health domain (7 items), Psychological domain (6 items), Social relationships domain (3 items), and Environmental domain (8 items). This generates a profile of domain scores. The two additional items will be examined separately: the overall perception of quality of life and overall perception of health. Each item is scored on a Likert scale ranging from 1 to 5. The items ask the respondent “how much,” “how often,” “how completely,” “how good” or “how satisfied” she felt about different aspects of her life in the past 2 weeks. The mean score of the items within each domain is transformed linearly to a domain score scaled in a positive direction from 0 to 100, such that higher scores indicate higher quality of life [41]. The instrument has previously been translated into Norwegian, Urdu and Somali according to existing internationally accepted guidelines, and has shown satisfactory results regarding validity and reliability [42].

**Table 1:** The safety behaviors used in the study Safe Pregnancy

We will now ask you if you ever have taken actions to protect yourself.			
	No	Yes	Not applicable
Have you ever:			
… hid money?			
… hid extra set of house and/or car keys?			
… established a code with family, friends or others (contacting to them about something agreed upon beforehand to indicate you need help)?			
… asked the neighbour to call police if violence begins?			
… removed weapons (such as knives)?			
… told someone how things are at home?			
… stayed at a crisis shelter?			
… documented bruises or violent events (like taking pictures)?			
Have you ever made sure you had available			
… social Security Numbers (yours, his, children)?			
… passport/ID or other important papers (marriage license, birth certificates)?			
… your own bank account?			
… valuable jewellery?			
… a bag of extra clothing for you and your children?			
… an extra phone or sim card?			
… important phone numbers (police, crisis shelter, ambulance)?			

The secondary outcomes are:

- Composite Abuse Scale (CAS): CAS R-SF is a 15-item instrument that captures physical, sexual and psychological abuse and overall Intimate Partner violence (IPV) [43]. The CAS R-SF is based on the validated 30-item Composite Abuse Scale, which is widely used to assess women’s self-reported experience of violence in an intimate relationship [44]. The CAS R-SF was developed to improve the CAS regarding response burden, brevity and clarity [43]. Women will be asked 15 questions about different actions and have the possibility to answer: Has this ever happened to you? Yes/No. If yes, how often did it happened in the last 12 month: Not in the past 12 months, once, a few times, monthly, weekly, daily/almost daily (0 to 5 scale). Total scores for the CAS R-SF, ranging from 0 to 75, will be calculated by computing mean of past 12-month frequency of abuse and multiplying by 15. For the questionnaire to be valid, no more than 3 items (out of 15) items can be missing. Subscale scores will be calculated for the physical, sexual and psychological abuse in a similar manner.
- Edinburgh Depression Scale-5 short version: Eberhardt-Gran et al. developed and validated a short version in Norwegian of the original Edinburgh Depression Scale [45]. This instrument consists of 5 questions. Each question has four response options, ranging from 0 to 3. Thus, the total score has a minimum of 0 and a maximum of 15. The total score is calculated and then a cut-off score of ≥7 of more is used. A score of 7 or more is considered an indication of the presence of symptoms of depression [45].
- Childbirth experience: We will use the following question to assess childbirth experience:
  What do you think about the statements below? During my delivery:
    I felt safe and in good hands
    I had severe pain
    I did not get enough pain relief
- Obstetric and neonatal outcomes: We will compare differences in the proportion of women in the intervention group with the proportion of women in the control group for the following outcomes: use of epidural analgesia, spontaneous vs. operative birth, low vs. normal birthweight, breastfeeding vs. not breastfeeding.

### Analysis plan

The study will be conducted as an RCT where two groups will be compared, the group who received the intervention video and the group who received the control video. Characteristics of participants in each of the two groups will be summarized using means and standard deviations for continuous data and frequencies and percentages for categorical data. Characteristics of the women in the two different groups will be compared to check if the groups are balanced concerning background variables and possible confounders. To account for possible differences regarding the characteristics of the women participating in relation to socio-economic factors such as age, education, ethnic background, and economic status, data on these variables will be collected and can be used in multivariate analyses. We expect the randomization to ensure a similar distribution of medical and obstetric factors such as parity, gestational age at filling out the questionnaire, complication during pregnancy and BMI.

For continuous outcomes, we will fit linear multiple mixed effects models with the unit as a random effect and selected covariates (possible confounders) will be fitted to assess the possible effect of the intervention. Further, we will assess a possible effect of the MCHCs on the use of safety behaviours. If there are significant differences between the MCHCs, we will treat MCHC as a fixed covariate and thus adjust for possible confounding in the multiple model. If there is an interaction between a group (intervention vs control) and a MCHC, we will present stratified analyses. The 19 MCHCs will be categorized as follows: Either based on the size, grouping small (< 100 women a year), medium (100 to 300 women a year) and large centres (> 300 women a year). Alternatively, we will compare the health centres in Oslo city with those located outside of Oslo city.

### Missing data

Model based imputation of missing data will be performed when less than 20% of values is missing for any given variable.

Intention to treat analysis will be performed. All tests will be two-sided. *P*-values < 0.05 will be considered statistically significant.

### Data management

A data management contract will be made between each study site and the project management. The recruiting midwives will keep a register with a study number and all the identifiable data (name, social security number, phone number) for use during the follow up. This register will be locked up with access only available to the midwife and the project coordinator. Other data, collected on the tablet, will not be stored on the tablet itself, but securely transferred to a secured server and stored encrypted at the server. These data do not contain personal identifiable information and are therefore seen as anonymous data. The Department of Technology Systems at the University of Oslo will handle the data management.

### Process evaluation

The process evaluation will investigate the extent to which the intervention was delivered as intended, what worked and what did not work. Semi-structured, qualitative interviews will be performed with a purposively selected sample of midwives. The interviews will be audio recorded, transcribed and reviewed by the work package leaders for emerging themes, sub-themes and codes. Themes that emerge will be discussed in the research group. The same research process will be used to explore women’s experience of screening and the intervention. The process evaluation will follow the consolidated criteria for reporting qualitative research [COREQ] [46].

### Ethics, safety and security

The trial protocol was approved by the Regional Ethics Committee South/East in April 2017 (ref nr: 2017/358). The studies included in this project will follow the Helsinki Protocol (WMA Declaration of Helsinki at https://www.wma.net/) and the WHO guidelines for researching violence against women: Putting women’s safety first: Ethical and Safety Recommendations for Research on Domestic Violence against women [47]. A Data Monitoring Committee (DMC) will be established in order to ensure that the trial and data collection is conducted appropriately. The DMC will consist of experienced researchers in randomized trials and IPV.

A woman will not be asked to participate in the study unless she attends the clinic on her own. Eligible women will receive written information about the study by the recruiting and a written informed consent will be obtained from each participant upon recruitment by the midwife.

All participants will receive information about options in their community regarding violence. The community health services are equipped to care for women experiencing IPV who request and need help [23]. The study group will ensure that all midwives have an overview of routines, procedures and referrals and develop these documents if needed. The midwives will follow the guidelines regarding routine enquiry about violence as usual (23). The study should not add any risk for the women. On the contrary, the extra education provided to midwives at the start of the study should enable midwives to access the resources available in the health services. Questions about violence are sensitive, but studies have shown that women are in favour of inquiry for IPV in antenatal care [48, 49] and they report meaningfulness about their participation in studies that includes questions about sensitive topics [50].

## Discussion

The project aims to provide evidence on whether asking about violence and short video intervention on a tablet is effective and feasible for the prevention and limitation of IPV among women who attend antenatal care. We hypothesise that routine enquiry about IPV during pregnancy, in combination with information about IPV and safety behaviours has the potential to increase the use of these behaviours and may interrupt IPV. We also anticipate that the participating midwives will experience a greater competence and confidence in approaching the topic of violence and handling positive answers. Women will benefit from health professionals who enquire in an appropriate way about violence. The screening tool and videos that are developed can easily be implemented in other communities across the country and may with minor adjustments even be useful for Somali and Pakistani women outside of Norway.

Strength with this study is a large number of MCHCs that can ensure a population-based sample of women. The involvement of midwives to whom women may have or can build a trusting relationship is also a strength. The tablet-based intervention is designed to blind both the participants and the providers and can minimize performance and selection bias. The tablets support Audio Computer Assisted Self Interviews (ACASI) that tend to yield higher rates of IPV disclosure [51]. It is also shown that self-completion IPV screening is welcomed by women [52]. The user involvement study is a strength to gain a deeper knowledge about cultural differences and create a cultural sensitive intervention that is recommended in public health interventions [9].

This study has potential limitations: Women do not always disclose the true nature of IPV and the prevalence may be under-reported. Thus, to recruit 150 women with recent or current violence exposure can be a challenge. While it is shown that women would like the health provider to ask about IPV, they may not be ready to disclose [53]. The AAS gives us the possibility to include women with previous violence experiences, and it may be easier for women to admit previous violence even if they are experiencing current violence. Ideally, we would have asked the women several times, because study shows this will increase disclosure [54]. This will not be possible within the current study. When asking about violence at baseline, women are likely to consider the role violence has in their lives and this may affect the outcome. It is difficult to conduct a true randomized trial in this field. All women, despite being in the intervention group or in the control group need to be offered some relevant information regarding violence and referrals for ethical reasons [47].

## Abbreviations

AAS: Abuse Assessment Scale
CAS: Composite Abuse Scale
IPV: Intimate partner violence
MCHC: Maternal and child health centres
RCT: Randomised controlled trial
WHOQOL-BREF: World Health Organization Quality of Life – Bref

## References

[1] Garcia-Moreno C, Pallitto C, Devries K, Stöckl H, Abrahams N. Global and regional estimates of violence aganist women: prevalence and health effects of intimate partner violence and non-partner sexual violence. Geneva; 2013.

[2] James L, Brody D, Hamilton Z (2013). Risk factors for domestic violence during pregnancy: a meta-analytic review. Violence Vict.

[3] Hjemdal OK, Engnes K: Å spørre om vold ved svangerskapskontroll. [Asking about violence in antanatal care (in Norwegian)]. Oslo NKVTS;2009.

[4] Henriksen L, Schei B, Vangen S, Lukasse M (2014). Sexual violence and mode of delivery: a population-based cohort study. BJOG..

[5] Lukasse M, Schroll AM, Ryding EL, Campbell J, Karro H, Kristjansdottir H (2014). Prevalence of emotional, physical and sexual abuse among pregnant women in six European countries. Acta Obstet Gynecol Scand.

[6] Finnbogadottir H, Dykes AK (2016). Increasing prevalence and incidence of domestic violence during the pregnancy and one and a half year postpartum, as well as risk factors: -a longitudinal cohort study in Southern Sweden. BMC Pregnancy Childbirth.

[7] Jewkes R (2002). Intimate partner violence: causes and prevention. Lancet..

[8] Bifdir. Rapportering fra krisesentertilbudene 2017. Barne-, ungdoms- og familiedirektoratet; 2017. [Report form the crisis shelters 2017, Norwegian Directorate for Children, Youth and Family Affairs (in Norwegian)].

[9] Resnicow K, Baranowski T, Ahluwalia JS, Braithwaite RL (1999). Cultural sensitivity in public health: defined and demystified. Ethn Dis.

[10] Pallitto C, Garcia-Moreno C, Stoeckl H, Hatcher A, MacPhail C, Mokoatle K (2016). Testing a counselling intervention in antenatal care for women experiencing partner violence: a study protocol for a randomized controlled trial in Johannesburg, South Africa. BMC Health Serv Res.

[11] Brownridge DA, Taillieu TL, Tyler KA, Tiwari A, Ko LC, Santos SC (2011). Pregnancy and intimate partner violence: risk factors, severity, and health effects. Violence AgainstWomen.

[12] Alhusen JL, Ray E, Sharps P, Bullock L. Intimate partner violence during pregnancy: maternal and neonatal outcomes. J Women's Health. 2014. 10.1089/jwh.2014.4872 PMC4361157 25265285

[13] Henriksen L, Vangen S, Schei B, Lukasse M (2013). Sexual violence and antenatal hospitalization. Birth..

[14] Schei B, Lukasse M, Ryding EL, Campbell J, Karro H, Kristjansdottir H (2014). A history of abuse and operative delivery-results from a European multi-country cohort study. PLoS One.

[15] Sorbo MF, Lukasse M, Brantsaeter AL, Grimstad H (2015). Past and recent abuse is associated with early cessation of breast feeding: results from a large prospective cohort in Norway. BMJ Open.

[16] Hooker L, Small R, Taft A (2016). Understanding sustained domestic violence identification in maternal and child health nurse care: process evaluation from a 2-year follow-up of the MOVE trial. J Adv Nurs.

[17] Vangen S, Ellingsen L, Andersgaard AB, Jacobsen AF, Lorentzen B, Nyflot LT (2014). Maternal deaths in Norway 2005-2009. Tidsskrift for den Norske laegeforening.

[18] Kripos. Nasjonal drapsoversikt 2018 https://www.politiet.no/en/aktuelt-tall-og-fakta/tall-og-fakta/drapsoversikt/ Accessed 30 Feb. [The National Criminal Investigation Service. National Homicide statistics. 2018 (in Norwegian)].

[19] Devries Karen M, Kishor Sunita, Johnson Holly, Stöckl Heidi, Bacchus Loraine J, Garcia-Moreno Claudia, Watts Charlotte (2010). Intimate partner violence during pregnancy: analysis of prevalence data from 19 countries. Reproductive Health Matters.

[20] Vatnar SK, Bjorkly S (2010). Does it make any difference if she is a mother? An interactional perspective on intimate partner violence with a focus on motherhood and pregnancy. J Interpers Violence.

[21] Norwegian Directorate of Health: Nasjonal faglig retningslinje for svangerskapsomsorgen. [National guidlines, ananatal care (in Norwegian)]. Oslo;2005.

[22] O'Doherty LJ, Taft A, Hegarty K, Ramsay J, Davidson LL, Feder G (2014). Screening women for intimate partner violence in healthcare settings: abridged Cochrane systematic review and meta-analysis. BMJ [Clinical research ed].

[23] Norwegian Directorate of Health: Nasjonal faglig retningslinje for svangerskapsomsorgen - hvordan avdekke vold. [National guidlines, ananatal care – how to uncover violence (in Norwegian)]. Oslo; 2014.

[24] McFarlane JM, Groff JY, O'Brien JA, Watson K (2006). Secondary prevention of intimate partner violence: a randomized controlled trial. Nurs Res.

[25] Davies JM, Lyon E, Monti-Catania D (1998). Safety planning with battered women: complex lives/difficult choices.

[26] WHO (2013). Responding to intimate partner violence and sexual violence against women.

[27] Tiwari A, Leung WC, Leung TW, Humphreys J, Parker B, Ho PC (2005). A randomised controlled trial of empowerment training for Chinese abused pregnant women in Hong Kong. BJOG.

[28] Kiely M, El-Mohandes AA, El-Khorazaty MN, Blake SM, Gantz MG (2010). An integrated intervention to reduce intimate partner violence in pregnancy: a randomized controlled trial. Obstet Gynecol.

[29] Van Parys AS, Verhamme A, Temmerman M, Verstraelen H (2014). Intimate partner violence and pregnancy: a systematic review of interventions. PLoS One.

[30] Pandis N, Chung B, Scherer RW, Elbourne D, Altman DG (2017). CONSORT 2010 statement: extension checklist for reporting within person randomised trials. BMJ [Clinical research ed].

[31] Tønnessen M (2014). Frukbarhet og annen demografi hvor innvandrere og deres barn født i Norge.

[32] Ozaki R, Otis MD (2017). Gender equality, patriarchal cultural norms, and perpetration of intimate partner violence: comparison of Male University students in Asian and European cultural contexts. Violence Against Women..

[33] Byrskog U, Olsson P, Essen B, Allvin MK (2014). Violence and reproductive health preceding flight from war: accounts from Somali born women in Sweden. BMC Public Health.

[34] Rabin RF, Jennings JM, Campbell JC, Bair-Merritt MH (2009). Intimate partner violence screening tools: a systematic review. Am J Prev Med.

[35] Moonesinghe LN, Rajapaksa LC, Samarasinghe G (2004). Development of a screening instrument to detect physical abuse and its use in a cohort of pregnant women in Sri Lanka. Asia Pac J Public Health.

[36] Gidman J (2013). Listening to stories: valuing knowledge from patient experience. Nurse Educ Pract.

[37] Egge H, Kvellestad K, Glavin K. Immigrants’ experiences of pregnancy, childbirth and the postnatal period in Norway - a qualitative study. Nordisk Tidsskrift for Helseforskning. 2018; 10.7557/14.4295.

[38] McFarlane J, Malecha A, Gist J, Watson K, Batten E, Hall I (2002). An intervention to increase safety behaviors of abused women: results of a randomized clinical trial. Nurs Res.

[39] McFarlane J, Malecha A, Gist J, Watson K, Batten E, Hall I (2004). Increasing the safety-promoting behaviors of abused women. Am J Nurs.

[40] McFarlane J, Parker B, Soeken K, Silva C, Reel S (1998). Safety behaviors of abused women after an intervention during pregnancy. J Obstet Gynecol Neonatal Nurs.

[41] World Health Organization (1998). WHOQOL user manual.

[42] Hanestad BR, Rustoen T, Knudsen O, Lerdal A, Wahl AK (2004). Psychometric properties of the WHOQOL-BREF questionnaire for the Norwegian general population. J Nurs Meas.

[43] Ford-Gilboe M, Wathen CN, Varcoe C, MacMillan HL, Scott-Storey K, Mantler T (2016). Development of a brief measure of intimate partner violence experiences: the Composite Abuse Scale [Revised]-Short Form [CASR-SF]. BMJ Open.

[44] Hegarty K, Sheehan M, Schonfeld C (1999). A multidimensional definition of partner abuse: development and preliminary validation of the composite abuse scale. J Fam Viol.

[45] Eberhard-Gran M, Eskild A, Samuelsen SO, Tambs K (2007). A short matrix-version of the Edinburgh depression scale. Acta Psych Scand.

[46] Tong A, Sainsbury P, Craig J (2007). Consolidated criteria for reporting qualitative research [COREQ]: a 32-item checklist for interviews and focus groups. Int J Qual Health Care.

[47] World Health Organization (1999). Putting Women’s safety first: ethical and safety recommendations for research on domestic violence against women.

[48] Stöckl H, Hertlein L, Himsl I, Ditsch N, Blume C, Hasbargen U (2013). Acceptance of routine or case-based inquiry for intimate partner violence: a mixed method study. BMC Pregnancy Childbirth.

[49] Garnweidner-Holme LM, Lukasse M, Solheim M, Henriksen L (2017). Talking about intimate partner violence in multi-cultural antenatal care: a qualitative study of pregnant women's advice for better communication in South-East Norway. BMC Pregnancy Childbirth.

[50] Widom CS, Czaja SJ (2005). Reactions to research participation in vulnerable subgroups. Account Res.

[51] Klevens J, Sadowski L, Kee R, Trick W, Garcia D (2012). Comparison of screening and referral strategies for exposure to partner violence. Womens Health Issues.

[52] Taft AJ, Hooker L, Humphreys C, Hegarty K, Walter R, Adams C (2015). Maternal and child health nurse screening and care for mothers experiencing domestic violence [MOVE]: a cluster randomised trial. BMC Med.

[53] Chang JC, Cluss PA, Ranieri L, Hawker L, Buranosky R, Dado D (2005). Health care interventions for intimate partner violence: what women want. Womens Health Issues.

[54] Campbell J, García-Moreno C, Sharps P (2004). Abuse during pregnancy in industrialized and developing countries. Violence Against women.

